# Infant Cranial Deformity: Cranial Helmet Therapy or Physiotherapy?

**DOI:** 10.3390/ijerph17072612

**Published:** 2020-04-10

**Authors:** Josefa González-Santos, Jerónimo J. González-Bernal, Raquel De-la-Fuente Anuncibay, Raúl Soto-Cámara, Esther Cubo, José M. Aguilar-Parra, Rubén Trigueros, Remedios López-Liria

**Affiliations:** 1 Department of Health Sciences, Cavidito Research Team, Health Research Centre, University of Burgos, 09001 Burgos, Spain; mjgonzalez@ubu.es (J.G.-S.); jejavier@ubu.es (J.J.G.-B.); rscamara@ubu.es (R.S.-C.); ecubo@ubu.es (E.C.); 2 Department of Educational Sciences, Discondu Research Team, Educational Research Centre, University of Burgos, 09001 Burgos, Spain; raquelfa@ubu.es; 3 Department of Psychology, Hum 878 Research Team, Health Research Centre, University of Almería, 04120 Almería, Spain; 4 Department of Language and Education, University of Antonio de Nebrija, 28015 Madrid, Spain; 5 Department of Nursing Science, Physiotherapy and Medicine, Hum 498 Research Team, Health Research Centre, University of Almería, 04120 Almería, Spain; rll040@ual.es

**Keywords:** plagiocephaly, helmet, physiotherapy, intervention, treatment

## Abstract

*Objective*: To compare cranial helmet therapy (CHT) and physiotherapy (PT) for the effective treatment of positional plagiocephaly in infants in terms of improving functional recovery. *Methods:* This was a prospective cohort study involving 48 infants between 5–10 months of age with cranial deformities. The Cranial Vault Asymmetry Index (CVAI) and the Brunet–Lezine scale were calculated at the initiation of the study and after 40 treatment sessions. *Results*: The infants’ first assessment showed a delay in overall development areas with a global developmental quotient (DQ) (posture, coordination, sociability, and language) of 80.15. Although developmental improvements were observed in both groups in the Brunet–Lezine scale after treatment, the MANCOVA test showed no significant differences (F(5) = 0.82, *p* = 0.506, eta^2^ = 0.09). The CVAI reduced to 4.07% during the final evaluation in the cranial helmet group and 5.85% in the physiotherapy group without any significant differences between the two therapies (*p* = 0.70). *Conclusions*: No statistically significant differences were found between CHT and PT. After treatment, improvements from baseline measurements were observed in each of the readings of cranial deformity.

## 1. Introduction

Cranial deformities (plagiocephaly, scaphocephaly, and brachycephaly), relatively frequent among infants, are still a topic of significant medical interest. A study, for instance, has noted a considerable increase in the number of medical check-ups relating to occipital plagiocephaly [1]. 

Non-synostotic plagiocephaly represents an asymmetry of the skull due to mechanical forces applied in the uterus or during the postnatal period [2]. It is accompanied by facial asymmetry and altered ear position. Estimations of its incidence vary noticeably between 3% and 48% [3,4]. Some authors have even estimated the prevalence of posterior positional plagiocephaly between 18–20% in newborns, varying with age and decreasing at older ages [4,5]; the differences are often explained by diagnostic criteria. 

The literature reports long-term clinical repercussions arising from the most severe cranial deformities, though unrelated to brain functions, such as deformities affecting orthodontic health and temporomandibular joint syndrome [5]. Several studies have reported an association between the presence of plagiocephaly and the delays in cognitive, language and motor development, which can persist up to 36 months of age [6,7,8,9]. Their results emphasize two hypothesized and potentially interacting pathways: environmental positioning as the primary driver for motor delays and simultaneously for plagiocephaly or an underlying central nervous system dysfunction as a primary driver of motor and cognitive delays, with motor delays leading to limited mobility and plagiocephaly [6,7,8]. This is the reason why the plagiocephaly may be a ‘physical marker for developmental delay’ [6]. Likewise, other types of alterations, such as psychosocial disorders or ocular diseases in the form of exotropia, have also been noted. A recent study has proved that correction of this condition is, in most patients, achieved through the application of different types of therapies, such as the cranial helmet [10]. While others did not show significant spontaneous improvement in cranial deformation within a timeframe of 5 years [11].

Risk factors for plagiocephaly include external mechanical pressure on the incompletely mineralized skull bone (intrauterine narrowing, supine sleeping position), male gender, firstborn, limited passive neck rotation, lower activity level or musculoskeletal cervical imbalance [12,13,14]. An additional risk factor is pericerebral accumulations of cerebrospinal fluid, which have been identified in 50% of the children, as tested in neuroimaging studies [1]. Thus, pregnancy and the first 4 months after birth are the critical period of plagiocephaly [12], which could be avoided by prevention guidance [13,15]. Plagiocephaly may, therefore, be interpreted as an early warning sign of slight cerebral dysfunction, and early cranial deformity may lead to such dysfunction [16]. 

Though the effectiveness of mainstream treatments has been addressed in numerous studies, no clear consensus has emerged yet [6,11,17]. However, some authors have indicated a positive prognosis with conservative treatments comprising physiotherapy for postural correction (PT) and cranial helmet therapy (CHT) [14]. 

All of the studies under analysis [14,18] have been found to agree on the need for proper therapeutic criteria, initially with conservative treatment. 

There are limited available data to assess the efficacy of physiotherapy as a primary treatment for plagiocephaly or compared with other therapies, although it is reported to be effective in the earlier reduction of skull asymmetry [19,20].

The initial experience of CHT as an orthopedic treatment for infants pointed out improvements within 2–3 months, as noted in different studies [21,22]. The results showed a reasonable and less traumatic alternative to surgical treatment in patients with severe plagiocephaly and postural brachycephaly [23]. A study comparing the effectiveness of CHT with no other additional therapeutic intervention reported that CHT led to a significant decrease in asymmetry in cases of severe plagiocephaly [24]. 

In some studies, a dynamic cranial helmet improved the deformity and permitted acceptable growth of the cranium [1,25]. In this line, a systematic review on non-surgical treatment of cranial deformities showed that CHT helmet therapy may reduce skull asymmetry more effectively than postural correction training [26]. However, this conclusion may be tempered due to potential biases in selected studies, such as different therapeutics techniques, initiation of therapy at different ages, therapies with different durations, inconsistency in diagnostic criteria and different ways of assessing the severity level of cranial deformities. Other studies also clearly noted the absence of any effect on developmental disorders due to the application of orthopedic treatments [16]. 

There is no consensus on the age of the infant in which CHT should be applied, despite it being a key factor to consider during treatment. Some studies have insisted on the effectiveness of application of this therapy before a patient reaches six months of age [27,28,29,30]. However, other authors have concluded that helmet treatment is equally effective if it is initiated within the first 12 months of life, since 85% of cranial growth occurs during this time [31,32]. Even, if the cranial molding therapy is applied in infants older than 12 months, twice the treatment duration will be required for a similar amount of correction [32]. Several studies suggest that in the subgroup of infants presenting with moderate to severe positional plagiocephaly, CHT should be preferred over conservative therapy [33,34]. In this line, the Congress of Neurological Surgeons guidelines, published in 2016, recommends CHT for infants with persistent moderate to severe plagiocephaly after conservative treatment (repositioning or PT) or presenting at an advanced age [35].

The influence of reshaping the deformity in children with pre-existing neurological disorders is still unknown and is an area requiring further research. 

The hypothesis of the present study was that both interventions contribute to a greater functional recovery of cranial deformity; CHT being at least as effective as PT in correcting the initial level of developmental delay. This hypothesis was based on previously referenced studies. The objective of the present study was to evaluate the impact of cranial helmet and physiotherapy interventions on the development and cranial asymmetry in a sample of infants with cranial deformity.

## 2. Methods

This was a prospective cohort study including 48 infants with cranial deformity. 

### 2.1. Participants

The study population comprised 60 infants who attended the main rehabilitation center in Spain exclusively for cranial deformities and were consecutively recruited by the specialists. An initial questionnaire addressed to the parents and guardians screened out extremely premature infants, rare syndromes, and infants with chromosomal alterations. The stipulated sample selection criteria included normal conditions at birth, screening out children with fetal distress, premature and low-weight children (only admitting infants >1.600 g), and with adequate Apgar scores (≥7 per min and ≥8 at five min). Infants with micro- or macrocephalies, determined by head circumference, were also screened out of the study. 

The sample consisted of 60 infants from the city of Burgos and the surrounding province (Spain); 12 children either did not fulfill the inclusion criteria or did not provide written informed consent (in accordance with the Helsinki Declaration) before treatment. Finally, 22 patients used helmets and 26 babies received physiotherapy treatment. There were no patients who dropped out the treatment.

### 2.2. Instruments

Cranial vault asymmetry (CVA), also known as oblique diagonal difference or transcranial difference, is defined as the diagonal difference and is obtained by subtracting the smaller cranial diagonal from the larger cranial diagonal [18]. The Cranial Vault Asymmetry Index (CVAI) can be defined as the absolute value of the difference in cranial diagonals (CVA), divided by the smaller diagonal, and multiplied by 100 (normal: <3.5%; mild: 3.5–7%; moderate: 7–12%; severe: >12%) [10,23]. This index allows for a direct comparison between cranial deformities in infants with varying head sizes. Diagonals are measured from the anterio-lateral skull to the posterior skull, at the level of the greater equator of the skull. These diagonals are measured 30 degrees clockwise and counterclockwise from the mid-sagittal line. The change in CVAI was calculated as the final CVAI minus the initial CVAI. A completely symmetrical skull will have an index of 0%. The revised Brunet–Lezine scale, as described in *Early Childhood Psychomotor Development Scale* [23,36], is one of the tests most often used for evaluation of infant development, between the ages of 2 and 30 months. Posture developmental quotient (DQ), coordination DQ, sociability DQ, and language DQ were the four major developmental areas that were studied. These sub-scores are used to calculate global DQ. The normal value for global DQ varies between 80 and 120 (mean value = 100). Global DQ below 70 indicates developmental delay [36]. 

### 2.3. Procedure and Interventions

Differences in infant development during the course of the study were analyzed by treatment type: CHT versus PT at two points in time (baseline and after 40 treatment sessions). 

A standardized pediatric physical therapy intervention program was designed, which involved a combination of exercises and manipulative procedures to reduce positional preference, musculoskeletal disorders, and cranial deformity. Infants received 40 sessions of PT, for 60 min each, twice a week, over a period of five consecutive months. During the first 10 min of each PT session, exercises involving infants’ neck and upper body muscles were performed, preparing the infant for further motor development, so that musculoskeletal disorders, including functional plagiocephaly, could be minimized. The second part of the session aimed at reducing cranial deformity with a combination of four different manipulative techniques: craniosacral therapy, passive exercises, Bobath method, and postural treatment.

Infants in the CHT group were made to wear custom-made helmets by orthotists for 23 h a day until the treatment was finished. During this period of five months, regular evaluation by an orthotist and modification of the helmet was done as required to allow proper skull growth. Parents were given all additional information about the helmet therapy (how the helmet should be worn, cleaning the helmet, and general care) prior to its initiation. The helmet was developed from a three-dimensional image obtained via a Rodin 4D scanner, which enabled pinpoint accuracy in the evaluations. In all cases, the same brand of helmet was used, which was made by Institute Sant Joan (ISJ) orthotists. The compliance was registered daily by parents report and weekly by the physiotherapist and orthotists reviews.

The variations in anthropometric measurement and development of the infant, performed before and after both therapies, were measured using the Brunet–Lezine scale and the CVAI. Anthropometric measurements were made using calipers or CT images, and were extrapolated using standardized 2D digital photographs of frontal, sagittal, and transverse planes by the same physician to minimize bias. 

The investigation adhered to the principles of the Declaration of Helsinki (University of Burgos Bioethics Committee Approval IR12/2018). Written informed consent was obtained from the parents or guardians.

### 2.4. Data analysis

Descriptive statistics including mean ± standard and median deviations were calculated. Quantitative variables were compared using Student’s t-test, MANCOVA and MANOVA. A multivariate analysis was performed to assess whether the type of birth or gender influenced the benefits of the intervention. Data normality was confirmed using the Shapiro–Wilk test. A *p*-value < 0.05 was considered statistically significant. The statistical analysis was performed using SPSS statistical software package version 25.0 (IBM SPSS Inc, Chicago, IL, USA).

## 3. Results

The sample comprised 23 males and 25 females with a mean age of 6.28 months (SD = 3.68) in the PT group and 5.10 months (SD = 2.32) in the CHT group at the first evaluation and 11.28 months (SD = 5.46) in the PT group and 9.80 months (SD = 4.31) in the CHT group at the final evaluation. The type of birth, according to infants’ gender, was 8% (2) forceps, 56% (14) cesarean, 36% (9) vaginal in girls, and 30% (7) forceps, 30% (7) cesarean, 40% (9) vaginal in boys.

The CVAI at first evaluation was 10.69% (SD = 5.58) in the total sample, 9.62% (SD = 5.59) in the cranial helmet, and 11.59% (SD = 5.51) in the physiotherapy group (*p* = 0.228). During the final evaluation, this parameter was reduced to 4.07% (SD = 2.26) in the first group and 5.85% (SD = 3.60) in the second group. There were no significant differences between the treatment groups (*p* = 0.70).

The two groups of infants, one treated with physiotherapy and one with helmet therapy, were homogenous at the beginning of the study, with no significant differences between groups in terms of the assessment of development (Table 1). 

The results at the initial evaluation, measured using the Brunet–Lezine scale, showed a delay in overall development areas with a global DQ of 80.15. The area showing the maximum apparent development was social area (DQ 84.35), followed by the coordination area (DQ 80.17), and the language area (DQ 78.65); the area showing the maximum developmental delay was the motor area (DQ 78.03). Both groups showed statistically significant improvements from baseline, in each of the measures post-intervention.

Table 2 shows the differential development scores that were obtained between the initial and final evaluations in both groups using the Brunet–Lezine scale. The areas that improved the most were language (8.77), followed by visual-motor (7.15), and motor (6.49); the social score was the worst of all (4.08).

The MANCOVA test showed no significant differences (F(5) = 0.82, *p* = 0.506, eta^2^ = 0.09) between the treatment groups in terms of the differential scores between the initial and final evaluation, using the pretest of the global score as a covariate. Developmental improvements were observed in both groups.

In addition, a multivariate analysis was carried out to assess the influence of the type of birth and sex on the benefits of the intervention, with no statistically significant difference. The MANOVA inferential analysis established that the differences due to gender were not significant, neither with respect to the physiotherapy group, with a level of statistical significance (*p* = 0.895, F(5) = 0.32, Wilks Lambda = 0.91; η^2^ = 0.09), nor the helmet group (*p* = 0.947, F(5) = 0.22, Wilks Lambda = 0.92; η^2^ = 0.08). Further, the differences between the type of birth in the physiotherapy group (*p* = 0.918, F(10) = 0.44, Wilks Lambda = 0.77; η^2^ = 0.12), or the helmet group (*p* = 0.773, F(10) = 0.63, Wilks Lambda = 0.63; η^2^ = 0.20) were not significant. Therefore, the intervention is invariant by gender or type of birth, producing the same effects in all cases.

## 4. Discussion 

The results of the study showed that both treatment groups, CHT and PT, progressively improved the development quotients of the infants in the five months between the initial and final evaluation. Moreover, there were no significant differences between treatment types.

The literature reports one study, which shows significant differences in parents´ perception of the cranial shape between infants´ scores at diagnosis and after 1 year of treatment [37]. In this study, a definite improvement was reported, but complete resolution did not occur in most cases. Others have identified improvements to varying degrees with both the treatments, i.e., CHT and PT in 136 cases (86%) versus little or no change in 22 cases (14%) [1]. A recent study has analyzed the effects of the age and severity in the treatments outcomes for patients with deformational plagiocephaly. Its results suggest that younger infants, with less severe conditions, require a shorter treatment at the time, and the infant in the end has less sequelae or residual cranial deformation [38]. Therefore, when infants are treated at younger ages or prior to progression of the cranial deformity, better clinical outcomes are achieved.

On comparing CHT and PT, various authors indicated that cranial orthotic treatments should be accompanied by a rigorous program of physiotherapy [39]. 

Nevertheless, numerous studies have examined this issue with no clear consensus. For instance, Lutterodt and colleagues [40] questioned the results of the study by Steinberg and colleagues [41]. Conflicting opinions have been found in terms of differences between those who promote helmet therapy, believing its effectiveness is superior in some cases to the clinical benefits of physiotherapy; while on the other hand, there are authors who are more inclined at applying conservative therapies [40]. 

It is certainly advisable to consider both approaches as both have their place in the treatment of plagiocephaly [14,39]. Moderate deformities can be treated with postural alignment and physiotherapy protocols. Nevertheless, several deformities are corrected more quickly and effectively with cranial orthotics, as concluded by various studies [1,14]. 

The application of both interventions independently has proven effective in the treatment of plagiocephaly [2,28,34], although the number of studies comparing both techniques is very small [41]. 

In milder cases, where the diagnosis is made early, cranial deformation can be managed by stretching exercises and a regular prone position; in the most severe cases, the use of a helmet may be necessary. In both cases, primary care physicians and parents can benefit from that specific information. After treatment, success rates for the acceptable cranial form can reach 92% [14].

The authors believe that a longitudinal study holds great importance, as it helps in detecting potential learning problems and can propose interdisciplinary measures. A similar approach was also suggested by Steinbock [42], where no long-term differences were found in the longitudinal study carried out to analyze the development of children following helmet therapy and other therapies, although differences were observed at the initiation, above all in the motor area. The mean age of the children at the time of the completion of the questionnaire was 8.9 years (SD 3.8 years, range 5.0 to 18.5 years). However, 34% of the children and adolescents with cranial deformities were receiving learning assistance and 14% were attending special education classrooms.

The present study poses some limitations. The development of the study in a single-center and the use of only an anthropometric measure could raise questions about the generalization of the results. The measurements were operator-dependent since the same physician determined the exact point of the landmarks at each visit, took standardized 2D digital photographs of frontal, sagittal and transverse planes, and then extrapolated craniometric evaluation, in order to reduce the possible bias. 

Plagiocephaly in infants has been associated with a developmental delay that can last until school-age. In several studies, mild plagiocephaly is not associated with increased risk of general cognitive and academic difficulties at school-age. However, children with moderate to severe forms of plagiocephaly perform significantly lower on both cognitive and academic measures; close developmental monitoring is recommended for this group. These results suggest that moderate/severe plagiocephaly may be a marker for developmental risk [6,43]. For future research, it would be very interesting to include the follow-up of this cohort up to seven years, since it is at this time that the child reaches his greatest motor development, and also, determining the difficulties in learning during the school period of these children would be then possible [6,42]. 

## 5. Conclusions

No statistically significant differences were found between CHT and PT which showed improvements from baseline in each of the measures in cranial deformity after treatment. Therefore, the use of the type of treatment could depend on other variables, such as economic factors, age of the child, accessibility to physiotherapeutic techniques, and opinion of the parents. Due to the lack of control group, it is not possible to state if the improvement in the infant’s development is due to the intervention, perhaps a larger study may find statistical significance in the changes in CVAI between the groups, and the desire of a 7-year study to see what happens when these children reach grade-school age. The authors, however, advise the combination of both techniques, starting with physiotherapy and complementing it with the cranial helmet in patients with greater CVAI.

## Figures and Tables

**Table 1:** Scores in the initial and final assessment by the Brunet–Lezine scale depending on the type of treatment.

Ratio of Development	Type of Treatment	*N*	Mean	Standard Deviation	*T*	*p*	*d*
Posture DQ Pretest	Physiotherapy	22	80.23	14.30	1.12	0.267	0.32
	Helmets	26	75.41	15.23			
Posture DQ Postest	Physiotherapy	22	85.15	17.19	0.30	0.760	0.09
	Helmets	26	83.84	11.48			
Posture Coordination DQ Pretest	Physiotherapy	22	81.19	16.99	0.46	0.645	0.13
	Helmets	26	78.97	15.88			
Posture Coordination DQ Postest	Physiotherapy	22	88.65	17.90	0.64	0.523	0.19
	Helmets	26	85.77	11.90			
Sociality DQ Pre-test	Physiotherapy	22	80.61	19.69	−1.46	0.151	−0.42
	Helmets	26	88.77	18.72			
Sociality DQ Post-test	Physiotherapy	22	86.65	20.80	−0.71	0.483	−0.21
	Helmets	26	90.54	16.69			
Language DQ Pretest	Physiotherapy	22	76.65	17.46	−0.82	0.417	−0.23
	Helmets	26	81.02	19.47			
Language DQ Postest	Physiotherapy	22	86.50	19.24	−0.39	0.698	−0.11
	Helmets	26	88.54	16.64			
Global DQ Pretest	Physiotherapy	22	79.60	16.23	−0.27	0.792	−0.08
	Helmets	26	80.82	15.58			
Global DQ Postest	Physiotherapy	22	86.79	18.00	−0.11	0.914	−0.03
	Helmets	26	87.29	12.96			

**Table 2:** Increase in the ratio of development between the initial and final assessment of each treatment group, assessment by the Brunet–Lezine scale.

Ratio of Development	Type of Treatment	*N*	Mean	Standard Deviation	F	*p*	η^2^
Posture	Physiotherapy	22	4.88	11.60	1.26	0.268	0.03
	Helmets	26	8.39	9.79			
Coordination	Physiotherapy	22	7.46	11.40	0.83	0.827	0.01
	Helmets	26	6.79	9.27			
Language	Physiotherapy	22	9.84	11.65	0.42	0.520	0.01
	Helmets	26	7.52	13.13			
Sociality	Physiotherapy	22	6.03	11.97	1.66	0.204	0.04
	Helmets	26	1.77	10.73			
Global	Physiotherapy	22	7.19	10.08	0.07	0.786	0.01
	Helmets	26	6.46	8.08			
